# Rapid Extrication versus the Kendrick Extrication Device (KED): Comparison of Techniques Used After Motor Vehicle Collisions

**DOI:** 10.5811/westjem.2015.1.21851

**Published:** 2015-04-29

**Authors:** Joshua Bucher, Frank Dos Santos, Danny Frazier, Mark A. Merlin

**Affiliations:** * Rutgers University, Robert Wood Johnson Medical School, New Brunswick, New Jersey; † United States Navy, Newark, New Jersey; ‡ Robert Wood Johnson Emergency Medical Services, Newark, New Jersey; § Newark Beth Israel Medical Center, Newark, New Jersey

## Abstract

**Introduction:**

The goal of this study was to compare application of the Kendrick Extrication Device (KED) versus rapid extrication (RE) by emergency medical service personnel. Our primary endpoints were movement of head, time to extrication and patient comfort by a visual analogue scale.

**Methods:**

We used 23 subjects in two scenarios for this study. The emergency medical services (EMS) providers were composed of one basic emergency medical technician (EMT), one advanced EMT. Each subject underwent two scenarios, one using RE and the other using extrication involving a commercial KED.

**Results:**

Time was significantly shorter using rapid extraction for all patients. Angles of head turning were all significantly larger when using RE. Weight marginally modified the effect of KED versus RE on the “angle to right after patient moved to backboard (p= 0.029) and on subjective movement on patient questionnaire (p=0.011). No statistical differences were noted on patient discomfort or pain.

**Conclusion:**

This is a small experiment that showed decreased patient neck movement using a KED versus RE but resulted in increased patient movement in obese patients. Further studies are needed to determine if the KED improves any meaningful patient outcomes in the era of increased evidence-based medicine in emergency medical services.

## INTRODUCTION

A common complaint after traumatic injuries is neck or back pain.^1^ The primary concern of the pre-hospital provider in handling and transporting a patient with a potential spinal cord injury is prevention of further neurologic injury. This concern is legitimate as spinal cord injuries have the potential to occur after transit or during early management at the scene.^2^

It is estimated that 3% to 25% of spinal cord injuries occur after the initial traumatic insult, either during transit or early in the course of management.^3^–^8^ As many as 20% of spinal column injuries involve multiple non-continuous vertebral levels; therefore, the entire spinal cord is potentially at risk.^9^–^11^

**Figure 1:**
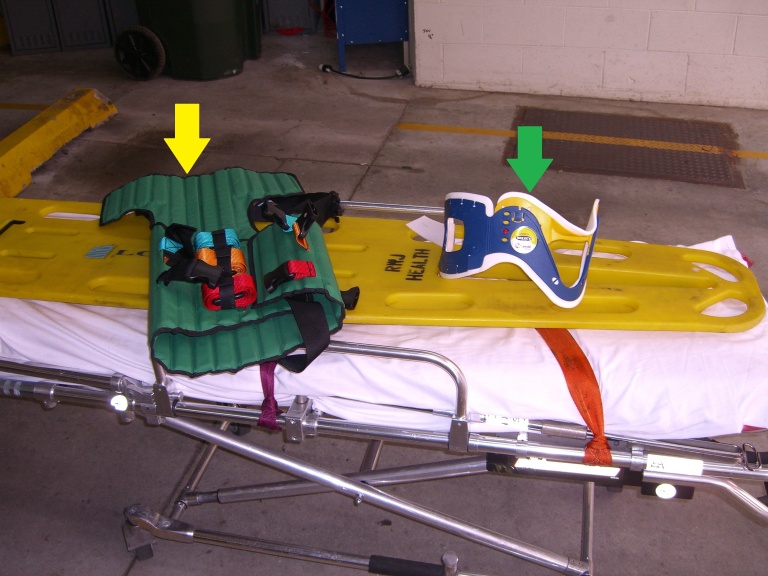
Application of Kendrick Extrication Device (yellow arrow) or cervical collar (green arrow).

Two common methods of immobilization in the pre-hospital setting include the Kendrick Extrication Device (KED) and rapid extrication (RE). The Kendrick Extrication Device (shown in Figure 1 with a yellow arrow) is used in the pre-hospital environment to stabilize patients complaining of neck or back pain after car collisions. The KED is a low-flexibility device that is secured to the patient’s torso, legs and head to prevent movement. It consists of three straps across the torso, an additional strap for the groin, and another strap that rides over the forehead. The back of the device is composed of several long blocks of hard, inflexible material with cloth in between to allow for flexibility related to the patient’s back.

RE is a method of moving a patient from the sitting to a supine position through a series of coordinated movements. Rapid extrication is indicated when the scene is unsafe, a patient is unstable, or a critical patient is blocked by another less critical patient. The standard longboard or backboard (shown in Figure 1, the large yellow device) is a device approximately six or seven feet in length that is hard and inflexible. The patient is secured to it using three or more straps and with two large foam blocks adjacent to the head referred to as cervical immobilization devices. RE involves immobilizing the patient on a longboard without the application of the KED.

For both devices, a c-collar (shown in Figure 1 with a green arrow) is applied while in-line stabilization is held. After application of the collar in a motor vehicle, the patient is either secured to a KED and removed or removed and secured to a longboard. The KED is most often used in motor vehicle crashes (MVCs) and other trauma involving back injuries such as falls.

The application of the KED may require significant movement of the patient in order to apply the device, causing further pain and possible further aggravation of the potential back/spinal injury. Furthermore, it places both the crew and the patient at risk due to operating under dangerous on-scene conditions for prolonged periods of time, such as on a highway with high speed traffic or in severe weather conditions.^12^,^13^ The FERNO KED manual states that, “The KED is designed for use by a minimum of two trained operators. Additional help may be preferred or needed.”^14^ This places an additional burden on the emergency medical services (EMS) crew to request additional trained personnel if necessary, which can be both time consuming and resource extensive.

There have been very few studies done on the KED. Graziano et al. determined, through radiographic imaging, that the KED was superior in reducing motion in all directions; however, that was compared to an older device no longer in use.^15^ Howell et al. determined, through radiographic imaging, that the KED superiorly limited rotational motion of the cervical spine but was similar in other planes to other immobilization techniques.^16^ Another study has found that the KED is an excellent device to use for the immobilization of pediatric patients, an off-label use.^17^ These studies evaluated movement of the cervical spine after application of the device, but not during the application process.

The goal of this study was to compare movement of the head, time to extrication and patient comfort for application of the KED versus RE by pre-hospital healthcare workers.

## METHODS

We used 23 subjects in 46 trials for this study. Subjects were included if they were over 21 and were able to give verbal consent to participate. We also excluded subjects if they were experiencing any pain prior to the beginning of the study.

Each rescue trial consisted of the participant and two EMS personnel. Both trials involved extricating the participant from a vehicle in a situation similar to a MVC by two EMS providers. The EMS providers were composed of one emergency medical technician (EMT), one paramedic. This was done to demonstrate consistency between each trial. All subjects underwent both scenarios.

Trial A involved a c-collar being applied, followed by the application of the KED, and extrication onto a longboard and ambulance stretcher. Trial B involved RE technique – a c-collar was applied and the participant extricated on to a longboard without a KED applied. The only difference between trial A and B was the use of the KED prior to extrication from the vehicle. The trial was time of arrival of EMS providers until the time the participant was correctly positioned on the ambulance stretcher, as determined by the researchers. We did not include securing the patients to the backboard. This would increase the time required to finish each trial but would not provide any additional information.

The angle of cervical spine movement was measured using a protractor placed on the bridge of the nose and a pen used to denote the plane of reference (a sagittal line). Subjects were asked to turn their head to the right and the left as far as tolerable. Angle measurement of movements was made at the following points in the KED group: after the KED has been applied and after the patient has been correctly positioned on the backboard. For the backboard-only group, the measurement of movement was made after c-collar application and after they had been correctly positioned on the backboard. The angle of measurement is axial movement, which was defined as asking the patient to turn their head to the left or right. Lateral rotation, which was not measured, is defined as moving the head laterally while maintaining the eyes forward. All trial scenarios were done with the seat in a standardized position of 19.75 inches from the tip of the steering wheel, and 120 degrees of steering wheel angulation. Seat belts were not worn during the scenarios.

We developed surveys to assess many variables associated with this study. The surveys were distributed to the participants after they have been extricated from the car and placed onto the stretcher.

**Figure 2:**
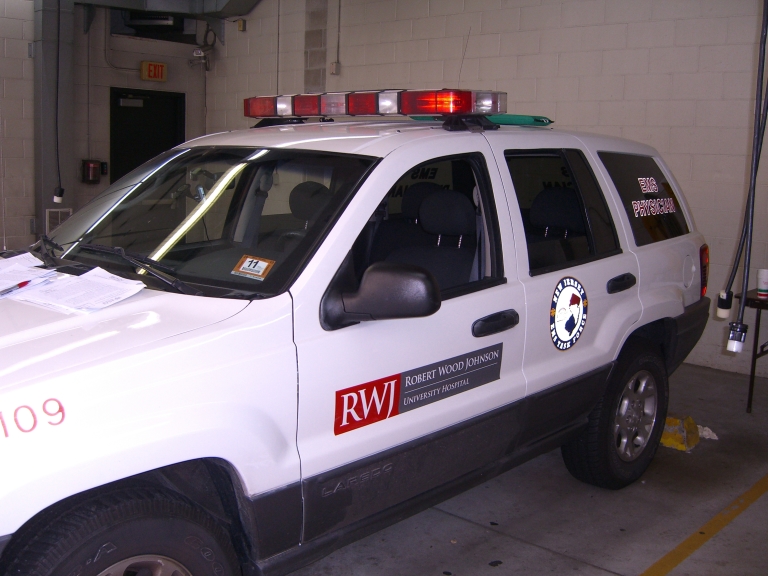
Vehicle used for study.

The participant surveys measured level of pain, level of discomfort, perceived amount of movement, and perception of amount of time taken to remove from car. These were asked at different stages: during application of KED or c-collar, and then extrication and positioning on the backboard (Figure 1). We measured these variables using a visual analog scale consisting of a 100mm horizontal line drawn with the two extremes of the variables at both ends. Participants were shown the line and the scale of 0–100 and asked to tell us what value they would like to ascribe to the specific question. This study was conducted in one of our EMS building garages using a 1995 Jeep Cherokee, which was fully functional and not damaged. Of the two person crew used in this study, one was an EMT instructor with 20 years of experience working approximately 40–50 hours per week who also worked for Robert Wood Johnson EMS as a paramedic (Figure 2). The other study participant was a volunteer EMT from a local volunteer first aid squad who also had over 10 years of experience.

This study received institutional review board approval at our institution, which has a subcontract with our hospital.

We conducted statistical analysis using SAS 9.1 TS level 1M0, XP_PRO platform (SAS Institute Inc., Cary, NC, USA) and MINITAB 15 (MINITAB Inc., State College, PA, USA).

We calculated summary statistics, including means, standard deviations and percentiles for times of extraction and degree of head turning for both conditions, extraction using KED versus RE.

Paired t-tests were used to examine basic differences in time and degree of head turning between these two techniques. We ran regression models to examine whether age, sex, height or weight modified the effect of the type of extraction. These regression models included the difference in outcome between KED and RE as the response variable and age, sex, height or weight as covariates. We repeated these analyses to summarize information about pain, comfort level and amount of movement experienced during the techniques.

## RESULTS

Table 1 provides summary statistics of the outcome variables, as well as p-values for detecting differences between outcomes under the two techniques.

Time was significantly different (shorter) using RE. In fact, there was no overlap in the times required by KED and RE (minimum KED time was greater than the maximum RE time). However, the angles of head turning were all significantly larger when using RE.

Weight marginally modified the effect of KED versus RE on the “angle to left after patient moved to backboard” (Table 2). Weight also significantly modified the subjective question about movement as heavier patients were associated with increased movement. There was a slight trend for patients in the heaviest weight category to experience either almost as much movement or more movement using KED than RE.

## DISCUSSION

Standards of care in the prehospital setting must be constantly reevaluated. Evidence-based care needs to be sought as many interventions in the pre-hospital environment have never been researched and have been based on anecdote.

The KED has been thought to improve spinal immobilization in patients complaining of traumatic induced neck or back pain. It has never been studied in live patients.

In our limited experiment, we found that extrication times are significantly shorter using RE versus KED. This is an important finding, as extrication of a patient from a vehicle is a time-consuming matter and may place the patient and the providers in danger due to environmental situations.

There was a notable difference in head turning with RE versus KED. This is not unexpected, as the KED does immobilize the head as securely as possible to the stretcher and backboard. Unexpectedly, a positive association with increasing weight and greater movement of the head to the left on RE versus KED was found in our study. This is likely due to the design of the devices, as neither device was designed for obese patients. There was no strong evidence for this finding due to a somewhat limited sample.

Subjects perceived a trend towards greater discomfort on the 100mm VAS with the KED versus RE. The heavier patient also perceived statistically significant more movement than less heavy patients on the KED. Both patients did perceive movement with the application of either device. The KED is supposed to be used on patients with neck and back pain after trauma. If the application of the device is causing greater movement of the patient, then the utility of this device should be called into question.

The KED can add considerable cost to an emergency medical service provider. FERNO charges $100 per device.^18^ This can become a considerable financial burden for EMS divisions. Also, parts must be replaced when destroyed. Sometimes, hospitals will cut the straps off instead of disconnecting the device properly. An EMS supplier website lists the replacement cost at $13.50 to replace all five straps.^18^ The KED is listed as a critical supply according to ambulance standards checklist and therefore must be carried on every ambulance.^19^ This can become a considerable financial burden for EMS agencies.

While this study provides limited data that the KED decreases ability of the patient to move their neck after application of the device, further studies are needed to determine if the device actually changes patient outcomes. In an era of increasing use of evidence-based care, all interventions that we commonly do based on anecdote need to be called into question. The National Association of EMS Physicians released a position paper last year on the use of longboards, as there is momentum to move away from longboards due to evidence that they can cause skin necrosis, worsen patient outcomes and have not been proven effective.^20^

## LIMITATIONS

There are several important limitations in our study. This was a single institution study where two EMS providers participated in each trial. Various EMS providers could have greater ability to use the KED or RE subsequently producing different results.

It is possible that different vehicles and angles of measurement could produce various results. We only measured axial movement and did not attempt to measure flexion, extension or lateral rotation. Axial movement was defined as asking the patient to turn their head to the left or right. Lateral rotation, which was not measured, is defined as moving the head laterally while maintaining the eyes forward.. This provides only limited information about total movement of the head during extrication. It should be noted that this was a controlled scientific experiment which is significantly different to performing the skill in the field with its more unpredictable variables. This was a controlled setting inside a garage with no risk for adverse weather or for suffering personal injury from vehicles on the road, which are frequently encountered when rendering pre-hospital care. Furthermore the vehicle was not damaged, which is not representative of most vehicular extrications. We also elected not to measure movement of the thoracic spine during our study. Measuring movement of the thorax would have been difficult to do using our study method.

Also, an expanded number of participants would enable more data to be collected and more significant analyses of the variables in the study.

## CONCLUSION

Based on our findings, we recommend that the utility of the KED needs to be further studied and compared to the rapid extrication technique. This study provides limited evidence for the use of the KED in patients who meet its indications that it can decrease their ability to laterally rotate their neck. It provokes concern with regard to using the device when prolonged scene time is a concern for provider or patient safety. KED’s beneficial effects are still largely unproven. Finally, there are additional concerns regarding the possible increased risk of movement of the spine in obese patients.

Further research should be conducted to determine whether the KED has a positive effect on patient outcomes and has any role in patient care.

## Figures and Tables

**Table 1:** Summary statistics for outcome variables using the KED versus RE on 23 subjects, as well as p-value for detecting a difference based on a paired t-test.

Variable	Technique	Mean (SD)	Minimum	Median	Maximum	Paired t-test p-value
Time (minutes)	KED	6.63 (1.29)	5.13	6.35	9.43	<0.0001
	RE	0.74 (0.26)	0.25	0.43	1.25	-
Angle to right after c-collar (RE)/KED applied (degrees)	KED	16.9 (9.0)	2	15	35	0.0028
	RE	24.1 (9.1)	10	25	45	-
Angle to left after c-Collar (RE)/KED applied (degrees)	KED	15.6 (9.2)	2	15	40	0.033
	RE	20.3 (9.1)	7	20	45	-
Angle to right after patient moved to backboard	KED	20.6 (11.5)	3	20	45	0.0025
	RE	30.8 (13.0)	15	30	70	-
Angle to left after patient moved to backboard	KED	21.6 (12.7)	3	20	50	0.045
	RE	26.9 (13.8)	3.0	25.0	55.0	-
Pain	KED	4.1 (8.0)	0	0	25.0	0.82
	RE	3.6 (10.0)	0	0	40.0	-
Discomfort	KED	25.7 (25.5)	0	20.0	70.0	0.11
	RE	16.5 (21.7)	0	10.0	75.0	-
Movement	KED	19.9 (26.4)	0	10.0	100.0	0.041
	RE	32.3 (31.8)	0	20.0	95.0	-

*KED*, Kendrick Extrication Device; *RE*, rapid extrication

**Table 2:** P-values of regression analysis testing significant effects of age, weight, sex, and height as modifiers of the effect of using KED versus RE.

	Modifying effect of			
	
Variable	Age	Weight	Sex	Height
Time	0.74	0.67	0.97	0.72
Angle to right after c-collar (RE)/KED applied	0.77	0.39	0.75	0.71
Angle to left after c-collar (RE)/KED applied	0.26	0.26	0.44	0.96
Angle to right after patient moved to backboard	0.23	0.029	0.38	0.16
Angle to left after patient moved to backboard	0.079	0.16	0.63	0.26
Pain	0.90	0.40	0.41	0.71
Discomfort	0.63	0.92	0.17	0.47
Movement	0.11	0.011	0.36	0.56

*KED*, Kendrick extrication device; *RE*, rapid extrication
